# Intimate partner violence and reduced dietary iron and vitamin A intake: a population analysis of nationally representative data from eight low- and middle-income countries

**DOI:** 10.1017/S1368980025000539

**Published:** 2025-04-14

**Authors:** Luissa Vahedi, Manuela Orjuela-Grimm, Pui Man (Pamela) Chan, Sarah R. Meyer

**Affiliations:** 1 Brown School, Washington University in St. Louis, St. Louis, MO, USA; 2 Factor-Inwentash Faculty of Social Work, University of Toronto, Toronto, ON, Canada; 3 The Centre for Global Child Health, SickKids Hospital, Toronto, ON, Canada; 4 Departments of Epidemiology and Pediatrics, Columbia University Irving Medical Center, New York, NY, USA; 5 Institute for Medical Information Processing, Biometry, and Epidemiology, Ludwig-Maximilians-Universität, Munich, Germany

**Keywords:** Demographic and health surveys, Minimum dietary diversity, Intimate partner violence, Dietary iron, Vitamin A

## Abstract

**Objective:**

This research provides the first population-based investigation of intimate partner violence (IPV) and women’s dietary intake of iron and Vitamin A-rich foods using representative data from eight low- and middle-income countries.

**Design:**

Using multivariable logistic regression, we estimated the relationship between various forms of past year IPV (physical, emotional and sexual) and consumption of Vitamin A and iron-rich foods.

**Setting:**

We conducted secondary data analysis of cross-sectional demographic and health surveys from Cambodia (2021, *n* 5640), Nepal (2022, *n* 4179), Sierra Leone (2019, *n* 3812), Nigeria (2018, *n* 8313), Tajikistan (2017, *n* 4800), Cote D’Ivoire (2021, *n* 3656), Kenya (2022, *n* 10 758) and the Philippines (2022, *n* 12 278).

**Participants:**

Women of reproductive age (15–49 years) comprised the analytical sample.

**Results:**

Results revealed distinct relationship patterns between various IPV forms and women’s dietary consumption of micronutrient-rich foods. The most consistent relationships being that past year (i) sexual IPV (adjusted OR (aOR): 0·72, 95 % CI: 0·53, 0·98), (ii) physical IPV (aOR: 0·86, 95 % CI: 0·73, 1·01) and (iii) emotional IPV (aOR: 0·81, 95 % CI: 0·70, 0·94) significantly *reduced* the odds of consuming iron-rich foods in the pooled analyses. Due to between-country heterogeneity concerning the relationship between IPV and Vitamin A, pooled estimates for dietary vitamin A consumption were non-significant. However, in the Philippines, IPV was associated with reduced dietary Vitamin A intake.

**Conclusions:**

IPV is associated with altered dietary intake patterns and between-country differences could be due to different food environments. Mechanisms explaining our findings may involve consequences of IPV that impact diet and dietary practices: depression, control of resources and physical trauma.

## Introduction

Intimate partner violence (IPV), defined as harmful behaviour perpetrated by a romantic partner/ex-partner, causing physical, sexual or psychological damage, is a highly prevalent global threat to well-being^(1)^. As one form of gender-based violence, the dynamics of IPV are rooted in the gender power hierarchy: while women do perpetrate IPV, the most prevalent, severe and chronic forms of IPV are perpetrated by men against women^(1)^. IPV is the most prevalent form of gender-based violence that women experience^(2)^. Recent global estimates indicate that 27 % of ever-partnered women between 15 and 49 years have experienced physical and/or sexual IPV in their lifetime, with 13 % of the same population reporting past year victimisation^(2)^.

IPV has significant and long-lasting impacts on women’s and children’s health. For example, IPV is associated with adverse mental health outcomes (depression, anxiety and suicidal ideation) and poor sexual health outcomes, such as HIV^(3)^. Maternal IPV also has adverse physical, mental and developmental impacts on children^(4)^. Research has begun to investigate linkages between gender-based violence (including IPV) and nutrition, focusing primarily on child nutrition outcomes^(5,6)^.

Nutrition refers to the nutrient intake required to maintain a healthy life at all stages. The WHO defines malnutrition as undernutrition, micronutrient deficiencies, overweight and obesity^(7)^. Malnutrition negatively impacts human development, reducing immune system functioning, increasing vulnerability to disease and limiting human potential and productivity^(8)^.

Essential micronutrients such as iron and vitamin A are key for health, and women are especially vulnerable to deficiencies in both. The mineral iron is crucial for oxygen transport, energy metabolism and cellular growth^(9)^, and its deficiency contributes to anaemia, with concomitant fatigue and cognitive impairment. Menstruation, pregnancy and lactation contribute to higher iron requirements in women of childbearing age^(9)^. Dietary Fe intake from Fe-rich foods is commonly insufficient and adherence to recommended Fe supplementation is poor, in large part due to gastrointestinal discomfort. Fe-rich foods thus become the primary sources of Fe for women. Fe deficiency during pregnancy increases the risk of preterm and low birth weight infants^(9)^. Vitamin A is a fat-soluble vitamin essential for vision, immune function, cell differentiation and fetal development^(10)^. Gestational deficiency of vitamin A can result in maternal night blindness and contributes to morbidity and mortality in early childhood^(10)^.

Understanding gendered risk factors for women’s reduced intake of Fe and vitamin A can strengthen existing violence protection and nutrition efforts that strive to improve women’s health and well-being. One gendered risk factor for inadequate nutrition may be women’s experience of IPV. Yet, there are significant evidence gaps concerning the relationship between IPV and women’s dietary nutrient intake. Analysing to what extent women’s experiences of IPV are associated with reduced diet quality can position IPV prevention and addressing survivors’ needs as actionable targets for nutrition actors. Some of the current evidence on women’s nutrition and IPV has focused on household food insecurity as a risk factor for IPV. For example, a mixed methods systematic review and meta-analysis of studies reported that food insecurity doubled the odds of violence against women and girls (including, but not restricted to, IPV)^(11)^. Longitudinal studies also robustly demonstrate that food insecurity increases IPV risk^(12)^. However, measures of food insecurity do not capture dietary patterns or nutrient intake and cannot examine how these may be affected following IPV.

A recent systematic review investigating the relationship between IPV and women’s nutritional status identified several knowledge gaps (Meyer et al., under review). Most published studies have focused on anthropometric indicators, suggesting an increased risk of inadequate BMI (both under and overweight) is associated with IPV^(13,14)^. A few studies suggested that IPV exposure was associated with an increased risk of anaemia^(14,15)^. Only three studies evaluated dietary intake: one study examined dietary diversity during pregnancy^(16)^, another study investigated habitual dietary adherence patterns during pregnancy^(17)^ and a third study examined select food group intake among college students^(18)^. Thus, only two studies used population-specific and valid measures of diet diversity^(16)^ or intake^(17)^ and none estimated nutrient content.

Measures of dietary diversity permit the examination of population level: (1) food group intake and (2) consumption of foods rich in specific nutrients, such as Fe and vitamin A. Using publicly available data from Demographic and Health Surveys (DHS) in eight diverse low- and middle-income countries (LMIC), we conducted a secondary analysis to examine relationships between women’s (15–49 years) experiences of past year IPV (physical, emotional, sexual) and their past 24-hour dietary intake of Fe and Vitamin A-rich foods. Given that the intrahousehold allocation of food can reflect gender power hierarchies^(19–22)^ and IPV can culminate in mental health symptoms/disorders^(23–27)^ and physical traumas^(28)^ that impact eating behaviours, we hypothesised that exposure to IPV would be associated with a lower likelihood of ingesting foods rich in Fe and Vitamin A.

### Method

#### Data sources and sampling

We analysed cross-sectional data from DHS conducted in eight countries: Cambodia (2021, *n* 5640), Nepal (2022, *n* 4179), Sierra Leone (2019, *n* 3812), Nigeria (2018, *n* 8313), Tajikistan (2017, *n* 4800), Cote D’Ivoire (2021, *n* 3656), Kenya (2022, *n* 10 758) and the Philippines (2022, *n* 12 278). These datasets met the following inclusion criteria: most recent DHS that was publicly available by October 2023 and included female participants interviewed with both the domestic violence module and the Minimum Dietary Diversity for Women tool. These inclusion criteria were needed to ensure the research team had access to the indicators (IPV and dietary intake) needed to answer the research question.

The DHS are nationally representative household surveys implemented in LMIC to monitor and evaluate important health and development indicators. Sampling for the DHS typically follows a two-stage cluster sampling procedure^(29)^. Prior to the first sampling stage, homogenous enumeration area subgroups (by sub-national and urban/rural regions) are created to allow for a representative sample to be drawn^(29)^. In the first stage of sampling, within each stratum, enumeration areas are randomly selected with a probability that is inversely proportional to the population size^(29)^. In the second sampling stage, about 25–30 households are randomly drawn from each enumeration area using equal probability systematic sampling^(30)^. Since the overall probability of selection for each individual is not constant, individual observations are weighted according to the population distribution of the national population by sub-national (for example, provinces) and urban/rural regions. Given the sampling strategy and weighing, estimates and inferences drawn from the DHS are nationally representative and collected using probability methods.

The DHS was implemented using computer-assisted personal interviews on tablets. For surveys conducted during the COVID-19 pandemic, protocols followed a COVID-19 risk mitigation plan. Overall existing survey reports indicated that the COVID-19 pandemic did not alter survey administration. However, in Cambodia, enumerators had to change routes or pause field activities due to outbreaks^(31)^ and in Cote D’Ivoire survey activities were suspended for a time^(32)^.

#### Exposures

The DHS measures IPV in the domestic violence module. The module follows the WHO guidelines on Ethical and Safety Recommendations for Research on Domestic Violence against Women^(33)^. Similar to the Conflict and Tactics Scale, the domestic violence module uses a list-based recall method of harmful behaviours that compromise IPV^(34)^. Before administering the domestic violence module, enumerators receive training on administration, dealing with crisis situations and how to prepare emotionally for this type of survey^(35)^. Informed consent is obtained prior to module implementation where the respondent is reassured of their confidentiality. Privacy is maintained by offering to reschedule the interview later when privacy can be maintained^(35)^. If there is an interruption in privacy, the module is stopped and resumed only when the other persons are out of hearing distance^(35)^. Referral services and other information are provided, in the form of an information sheet, to women who complete the module^(35)^.

In the DHS, one woman per household is randomly chosen among all eligible women within households selected for the individual questionnaire corresponding to this module^(36)^. The number of households selected for the Domestic Violence Module differs in each country. For example, in Nigeria, one-third of the household sample was administered the module^(37)^; in Nepal^(38)^ and Sierra Leone,^(39)^ half of the selected households were administered the module and in Kenya,^(40)^ three-quarters of the household sample were administered the module. The Domestic Violence Module weight is applied to the analysis to account for the random selection of households and/or women and nonresponse, thereby ensuring that the Domestic Violence Module subsample is nationally representative. External sources for the Domestic Violence Module are also available in the online supplementary material, Supplemental Appendix A.

Women who reported ever having an intimate partner (husband or boyfriend) were queried about past 12-month IPV experience using questions corresponding to physical (seven items), emotional (three items) and sexual (three items) IPV. We constructed binary variables for exposure to each of the three forms of IPV in the past 12 months (See Table 1). While severe forms of past year IPV (for example, those engendering life) can be disaggregated, limited sample sizes precluded us from doing so.

**Table 1.** Variable operationalisation strategy

Variables	Survey questions	Operationalisation
Exposures		
Past 12-month physical IPV (binary)	Physical IPV was measured by asking women to report whether a current boyfriend/husband perpetrated the following acts within the past 12 months: (1) pushed, shook or threw something; (2) slapped; (3) twisted your arm or pulled your hair; (4) punched you with a fist or with something that could hurt you; (5) kicked, dragged or beat you up; (6) tried to choke or burn you on purpose; or (7) threatened or attack you with a knife, gun or any other weapon^(30)^	Women who experienced at least one harmful act of physical IPV in the past 12 months were coded as exposed. The referent group included all women ever in a romantic relationship who did not report any physical IPV in the past year
Past 12-month sexual IPV (binary)	For sexual IPV, respondents were asked if in the past 12 months, they had been ‘(1) physically forced to have sexual intercourse even when they did not want to, (2) physically forced to perform any other sexual acts they did not want to or (3) forced with threats or in any other way to perform sexual acts they did not want to’ by a husband/boyfriend^(30)^	Women who experienced at least one harmful act of sexual IPV in the past 12 months were coded as exposed. The referent group included all women ever in a romantic relationship who did not report sexual IPV in the past year
Past 12-month emotional IPV (binary)	For emotional IPV, respondents indicated whether in the past 12 months they experienced a partner ‘(1) say or do something to humiliate you in front of others, (2) threaten to hurt or harm you or someone close to you or (3) insult you or make you feel bad about themselves’^(30)^	Women who experienced at least one harmful act of emotional IPV in the past 12 months were coded as exposed. The referent group included all women ever in a romantic relationship who did not report any emotional IPV in the past year
Outcomes		
Consumption of vitamin A-rich foods (binary)	Now I would like to ask you about (other) liquids or foods that you may have had yesterday during the day or at night. I am interested in whether you had the item even if it was combined with other foods^(41)^: Milk such as tinned, powdered or fresh animal milk? Tea or coffee? Any other liquids? Bread, rice, noodles or other foods made from grains? Pumpkin, carrots, squash or sweet potatoes that are yellow or orange inside? White potatoes, white yams, manioc, cassava or any other foods made from roots? Any dark green, leafy vegetables? Ripe mangoes, papayas or (locally available vitamin A-rich foods)? Any other fruits or vegetables? Liver, kidney, heart or other organ meats? Any meat, such as beef, pork, lamb, goat, chicken or duck? Eggs? Fresh or dried fish or shellfish? Any foods made from beans, peas, lentils or nuts? Cheese, yogurt or other milk products? Any oil, fats or butter or foods made with any of these? Any sugary foods such as chocolates, sweets, candies, pastries, cakes or biscuits? Any other solid or semi-solid food?	Foods identified by FAO as being rich in Vitamin A include rich fruits and vegetables that are yellow or orange on the inside. We operationalised the consumption of Vitamin A-rich foods as binary: women who reported consumption (in the past 24 h) of at least one Vitamin A-rich food and women who did not^(41)^
Consumption of Fe-rich foods (binary)		According to the FAO, foods that are rich in Fe include meat organ meat, poultry, fish/seafood and dark green leafy vegetables^(41)^ We operationalised the consumption of Fe-rich foods as binary: women who consumed at least one Fe-rich food source and women who did not consume any Fe-rich food sources

#### Outcomes

The Minimum Dietary Diversity for Women (MDD-W) tool was designed to capture population-level dietary pattern indicators and has been validated for women aged 15–49 years^(41)^. The tool includes documentation regarding the past 24 h intake of food items belonging to ten specific food groups. The DHS uses a list-based recall by asking women to report whether, on the prior day, they had consumed foods from a specific list organised by food categories (refer to Table 1 for a generic version of the MDD-W food items). These food items can be further classified into ten groups : (1) grains, starches, (2) beans, peas, lentils, (3) nuts, seeds, (4) milk, milk products, (5) meats (organs, meat, poultry, fish, seafood; intake of sausages and other processed meats was queried only in select countries and thus could only be included for Nepal, Cambodia, Cote D’Ivoire, Kenya and Philippines), (6) eggs, (7) dark green leafy vegetables, (8) vitamin A-rich fruits, vegetables, roots, tubers, (9) other vegetables and (10) other fruit. The individual food items were adapted for each DHS based on country-specific population habits. Table 2 displays the specific food items, corresponding to Fe-rich and Vitamin A-rich foods, that were queried in each country. We constructed two binary outcomes related to women’s nutrient intake reported for the 24 h prior to the DHS survey: presence or absence of (1) consuming at least one Fe-rich food item derived from plant or animal sources and (2) consuming at least one vitamin A-rich fruit or vegetable ^(41)^ (Refer to Table 1). External sources detailing the MDD-W are also available in the online supplementary material, Supplemental Appendix A.

**Table 2.** Fe-rich and vitamin A-rich food items queried, by Demographic and Health Surveys country and year

Country and data collection period	Food items queried that were included in Fe-rich food sources	Food items queried that were included in vitamin A-rich food sources
Cambodia September–February 2022	Sweet leaf bush (slek ngob), cassava leaves, spinach, tree spinach (chaya), kale or wild greens (slek prech)? Liver, kidney, heart, lung or blood? Sausage or ham? Any other meat, such as beef, buffalo, pork, frog, Fish (trey), seafood, eel, small shrimp (kompers), canned fish or fermented fish (pa’ork)?	Ripe mango, ripe papaya or passion fruit? Carrot, pumpkin or sweet potato that is yellow or orange inside?
Nepal January–June 2022	Saag, spinach, mustard greens, fennel greens, pumpkin shoots, taro leaves or amaranth greens, gundruk, braised greens, fenugreek greens or broccoli? Liver or organ meat? Sausages, ham, bacon or canned meat? Any other meat, such as goat, mountain goat? Fish or dried fish?	Carrots or ripe yellow pumpkin? Papaya, ripe mango, apricot or persimmon?
Nigeria August–December 2018	Ugu, bitter leaf (ewuro/onugbu), zogale (moringa), yakuwa (sorrel leaves), soko, ewedu/ayoyo, afang/okazi, sweet potato leaves, cassava leaves, cocoyam leaves, amaranthus/spinach (green/tete), water leaf, oha leaf, karkashi, kuka (baobab, luru), lansir, yadiya, rama, tafasa, kanya, cress, lettuce, yanrin (wild spinach), eku gogoro, eku petere, ilasa (young okro leaves), igbagba, ebolo, atama, editan, scent leaf (ntong/nchuawu/ arigbe/aluluisi), chaya (iyana paja), egg plant leaves? Liver, kidney, heart, gizzard? Meat, chicken and other bush meat/bird, kundi, kilishi, dambu nama, ponmo (cow skin)? Fish, crab, lobster, cray fish, shrimp, stock fish (okporoko)?	Ripe pawpaw (gwanda/ibeppe/okwuruoru/bobo), ripe mango, ripe passion fruit, dorowa (locust bean fruit), red palm fruit, hog plum (tsadan gida, iyeye, ngulungu), ripe cantaloupe, musk melon, monkey cola (ndiya), bush mango fruit (ugili/ogbono/mbupauyo)? Squash that is orange inside, pumpkin, carrot, red sweet pepper (tatase), sweet potato that is orange inside (orange flesh sweet potatoes)?
Sierra Leone May–August 2019	Bitter leaf, Moringa, Sorrel leaves, sweet potato leaves, cassava leaves, cocoyam leaves, amaranthus/spinach, water leaf, lettuce, wild spinach, young okro leaves, eggplant leaves, other green leaves eaten? Liver, kidney, heart, gizzard? Beef, mutton, goat, rabbit, chicken, goose, turkey, quail, pork, lamb, grass cutter, guinea fowl, hawk, monitor lizard, pigeon, small kangaroo, dove, squirrel, guinea pig, deer, alligator lizard, crocodile, peacock, camel, antelope, bat, bush rat and other bush meat/bird, horse, camel, duck, ox tail, cow leg, cow skin, biscuit bones, lung, stomach, intestines, tongue, brain, spleen, frog, toad, porcupine, dog, monkey, snake? Fresh fish, frozen fish (e.g. mackerel/Titus), canned fish (sardine, Geisha), smoked fish, dried fish, crab, lobster, crayfish, shrimp, stock fish, bonga fish, mudfish, tilapia, catfish, barracuda, any other type of fish?	Squash that is orange inside, pumpkin, carrot, red sweet pepper, sweet potato that is orange inside (orange flesh sweet potatoes), cassava? Ripe pawpaw, ripe mango, ripe passion fruit, locust bean fruit, red palm fruit, hog plum, ripe cantaloupe, musk melon, monkey cola, bush mango fruit?
Tajikistan August–November 2017	Any dark green, leafy vegetables (spinach, dark green lettuce, beet leaves)? Liver, kidney, heart or other organ meats or blood-based foods, including wild game? Any meat, such as beef, lamb, goat, pork, rabbit, wild game meat, chicken, turkey, duck or other bird? Fresh, canned or dried fish, caviar, squid, shrimp, crabs or any other seafood?	Sweet red bell pepper, pumpkin or carrots that are yellow or orange inside? Ripe persimmons or ripe fresh apricots, dried apricots or dried peaches or other fruits that are dark yellow or orange inside?
Cote D’Ivoire September–December 2021	Cassava leaves, spinach, potato leaves, kplala, dah leaves, taro leaves or leaf sauce? Offal (guts/intestines/guts/stomach/heart/liver/kidney) Paté, sausage, canned meat or ham? Other meats such as beef, mutton, frog Fresh fish, smoked fish, magne, box of sardines, crab or shrimp?	Carrot, pumpkin, squash or sweet potato with orange flesh? Ripe mango, ripe papaya, passion fruit?
Kenya February–July 2022	Sukuma wiki, spinach, managu (nightshade), terere (amaranth), saget or kunde (cowpea leaves), Khandira (Ethiopian kale), mrenda (jute mallow), pumpkin leaves, nderema (Malabar spinach), mitoo, broccoli or mchunga? Liver, blood, kidney, lung, gizzard or heart? Sausages, Smokies, hot dogs, salami or ham? Any other meat, such as goat, beef, minced beef, mutton, pork, wild game or chicken? Fish, dagaa, canned tuna or seafood?	Carrots, pumpkin, butternut or sweet potato that is orange inside? Ripe pawpaw, ripe mango, passionfruit or matunda ya damu?
Philippines May–June 2022	Moringa leaves, Chinese cabbage, camote leaves, water spinach, sayote leaves, yam leaves or bitter gourd leaves? Dinuguan, liver, heart, kidney or gizzard? Hot dogs, sausages, longganisa, chorizo, canned meats, tocino or tapa? Any other meat, such as beef, goat, pork, chicken or duck? Fish, sardines, daing or tuyo, dilis, smoked fish or seafood?	Carrots, squash or orange camote? Ripe mango, ripe papaya, orange-colored melon or chiesa?

#### Covariates

To determine the appropriate set of covariates for the adjusted logistic regression models, we developed a directed acyclic graph based on existing literature. Only variables that confounded the relationship between gender-based violence and women’s dietary micro-nutrient intake were included in the analysis as covariates (see online supplementary material, Supplemental Appendix B for the directed acyclic graph). The final set of confounders we included were: household wealth (ordinal), women’s education (ordinal), women’s age (numerical), husband’s degree of alcohol consumption (ordinal), presence of a polygynous household (binary), current pregnancy and/or breastfeeding status (binary), number of live births (numerical), presence of children under 2 years (binary), presence of children between 2 and 4 years (binary), presence of children between 5 and 9 years (binary) and presence of children 10–13 years (binary). Refer to online supplementary material, Supplemental Appendix C for the full covariate operationalisation strategy.

#### Statistical analysis

All data analysis was performed using Stata/se 17.0. To capture women (15–49 years) currently at risk of IPV who were living with their abusers and consuming food within the same household, we restricted the analytical sample to women who reported they were currently in an intimate relationship (marriage or cohabitation) at the time of the interview. Analyses were performed using sampling weights (corresponding to the Domestic Violence Module), primary sampling units and strata. First, descriptive statistics were computed for descriptive and demographic variables, by country. We computed means for numerical variables and proportions for categorical variables as well as their corresponding 95 % CI.

Second, we used generalised linear regression models (refer to online supplementary material, Supplemental Appendix E for the adjusted models). Adjusted logistic regression models were constructed for each dietary outcome as a function of past year physical, emotional, sexual or any IPV. We visually presented the results of the statistical models using forest plots. The pooled OR and 95 % CI for each exposure and outcome combination were computed using random effects models, given the heterogeneity between countries.

We also developed a strategy for post hoc sensitivity testing (refer to online supplementary material, Supplemental Appendix F). We considered the consumption of only animal protein sources for the Fe outcome. We also assessed the relationship between self-reported IPV and household wealth to investigate context-specific IPV patterns. Lastly, in Sierra Leone, we noted contradictory findings relative to our hypothesis; we ran competing models for the Vitamin A models where starchy staples were included as the outcome. This sensitivity model for Sierra Leone was also stratified by household wealth (as opposed to household wealth being included as a covariate).

### Results

#### Descriptive statistics

Descriptive statistics for each country included in the analysis are presented in Table 3. Reported experiences in the past year of physical IPV ranged from 3 % in the Philippines to 39 % in Sierra Leone. The range for reported emotional IPV was 7 % in the Philippines to 39 % in Sierra Leone. Past year sexual IPV was lower, ranging from 1 % in the Philippines and Tajikistan to 8 % in Kenya. Women’s mean ages ranged from 31 to 35 years. The average number of live births ranged from a minimum of about 2 in Nepal and Cambodia to a maximum of about 4 in Nigeria. The proportion of women consuming at least one Fe-rich food source (both animal and plant sources) was the lowest in Nepal and Kenya (76 %) and Tajikistan (83 %). The proportion of women consuming at least one Vitamin A-rich fruit or vegetable was highest in Sierra Leone (63 %) and the Philippines (60 %) and lowest in Cote D’Ivoire (15 %) and Nepal (22 %).

**Table 3.** Descriptive statistics among women (15–49 years) who reported being currently in a relationship and responded to the domestic violence module, using the nationally representative demographic and health surveys

	Sierra Leone (2019)			Tajikistan (2017)			Nigeria (2018)			Nepal (2022)			Kenya (2022)			Cote D’Ivoire (2021)			Cambodia (2021)			Philippines (2022)		
Variables	%		95 % CI	%		95 % CI	%		95 % CI	%		95 % CI	%		95 % CI	%		95 % CI	%		95 % CI	%		95 % CI
Physical IPV (past year)	39·06		36·59, 41·54	18·26		16·03, 20·49	11·48		10·45, 12·51	12·15		10·75, 13·55	18·27		17·13, 19·41	11·00		9·62, 12·39	4·37		3·64, 5·10	3·37		2·85, 3·89
Emotional IPV (past year)	39·07		36·67, 41·47	12·74		11·02, 14·46	27·00		25·60, 28·39	10·59		8·99, 12·20	25·68		24·44, 26·92	19·89		17·98, 21·81	12·70		11·55, 13·85	6·69		6·01, 7·38
Sexual IPV (past year)	6·20		4·94, 7·47	1·31		0·86, 1·76	4·56		3·96, 5·17	4·38		3·65, 5·11	7·76		6·93, 8·59	4·07		3·25, 4·88	1·88		1·36, 2·39	1·37		1·06, 1·67
Currently pregnant and/or lactating	38·34		36·51, 40·17	32·57		30·94, 34·19	49·34		48·05, 50·63	26·80		25·22, 28·39	37·43		36·03, 38·83	40·91		38·98, 42·85	20·96		19·62, 22·30	20·13		19·05, 21·22
Polygynous couples	27·44		25·60, 29·27	2·69		2·14, 3·23	20·73		19·45, 22·02	2·13		1·61, 2·65	3·03		2·63, 3·43	15·84		14·25, 14·25	n.a		n.a	n.a		n.a
	µ	s d		µ	sd		µ	sd		µ	sd		µ	sd		µ	sd		µ	sd		µ	sd	
Age (in years)	32·04	0·17	31·70, 32·38	32·18	0·16	31·88, 32·49	31·24	0·10	31·03, 31·44	32·46	0·16	32·14, 32·78	31·87	0·12	31·62, 32·11	31·48	0·20	31·1, 31·85	33·64	0·15	33·35, 33·93	35·25	0·11	35·03, 35·47
Number of live births	3·58	0·05	3·49, 3·67	2·82	0·03	2·75, 2·89	3·81	0·04	3·74, 3·89	2·23	0·03	2·17, 2·29	2·94	0·03	2·88, 3·00	3·36	0·05	3·25, 3·46	2·24	0·03	2·19, 2·29	2·50	0·03	2·45, 2·55
Highest year of completed education	3·18	0·10	2·98, 3·37	10·27	0·07	10·13, 10·41	6·42	0·12	6·19, 6·65	5·53	0·12	5·29, 5·77	8·64	0·07	8·5, 8·77	2·64	0·12	2·37, 2·88	5·76	0·09	5·58, 5·95	11·29	0·09	11·12, 11·46
Consuming Fe-rich foods	95·00		94·10, 95·89	83·34		81·80, 84·88	89·36		88·44, 90·27	75·89		74·07, 77·70	75·80		74·69, 76·91	89·15		87·55, 90·76	98·76		98·45, 99·07	96·64		96·14, 97·14
Consuming vitamin A-rich foods	62·52		59·65, 65·39	61·14		58·69, 63·59	30·31		29·00, 31·63	21·96		19·86, 24·06	27·35		25·83, 28·86	15·06		13·10, 17·03	39·10		37·21, 40·99	59·57		58·03, 61·11

Note: All descriptive statistics account for the complex sampling design and weighting. ‘n.a’ corresponds to ‘not applicable’ because the select variable was not included in all Demographic and Health surveys. ‘µ’ represents the population mean and sd represents the se for count and continuous variables, and % represents the population proportion (considering the weights and complex survey data). IPV, intimate partner violence.

#### Relationship between intimate partner violence and intake of foods rich in select micronutrients (iron and vitamin A)

**Figure 1.**
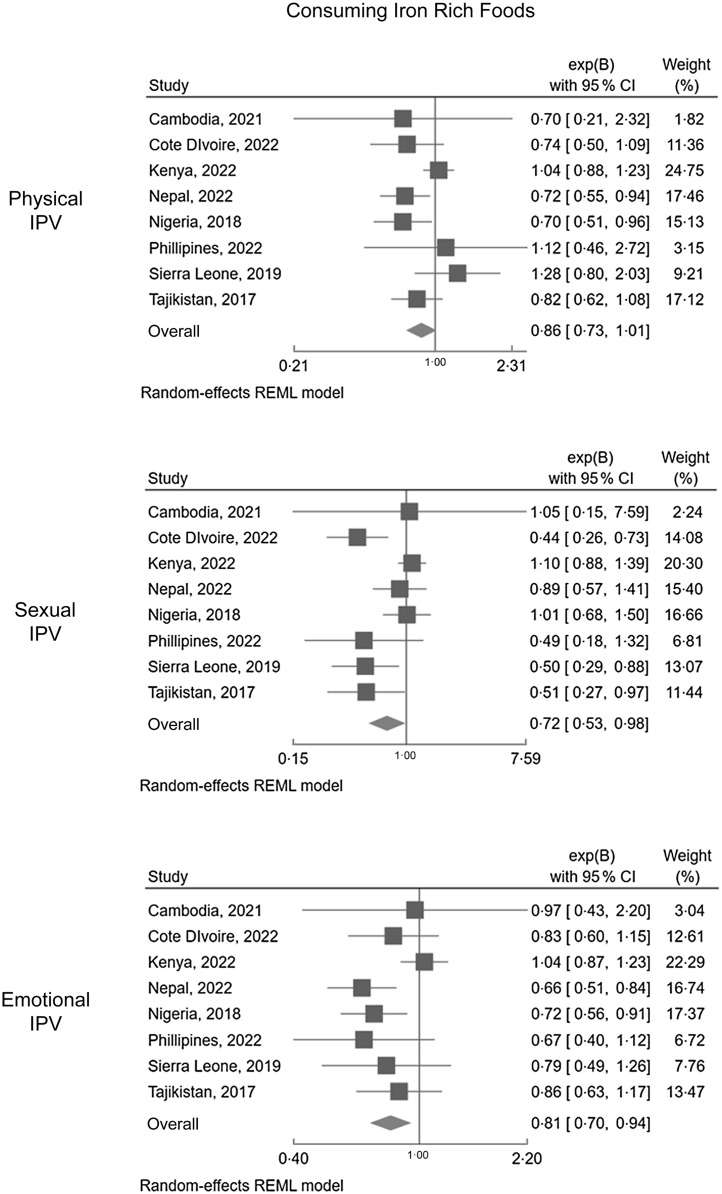
Forest plots displaying associations between IPV (physical, sexual, emotional) and dietary Fe consumption.

**Figure 2.**
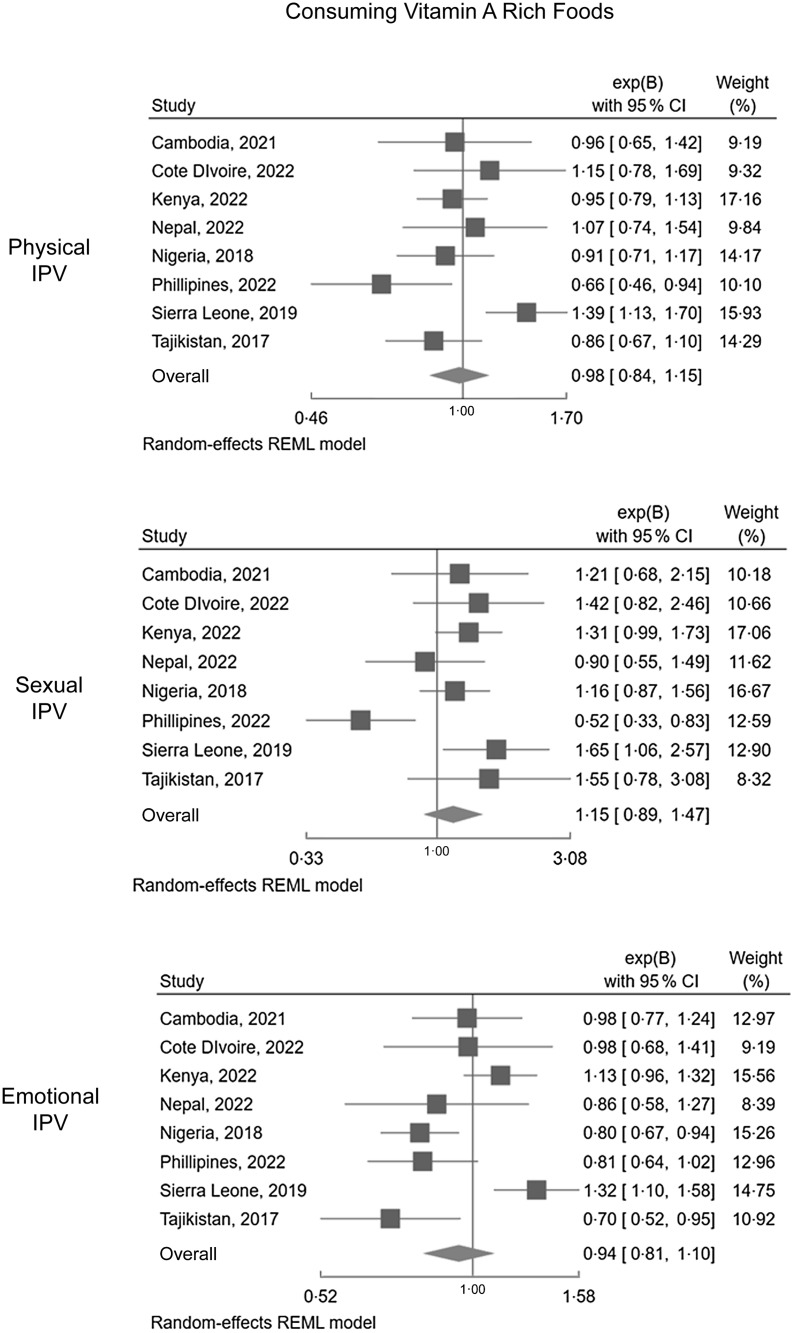
Forest plots displaying associations between IPV (physical, sexual, emotional) and dietary Vitamin A consumption.

Associations between the three forms of past year IPV (physical, emotional and sexual) and: (i) dietary Fe intake are presented in Figure 1 and (ii) vitamin A in Figure 2. Online supplementary material, Supplemental Appendix D contains the table format of Figure 1 and Figure 2.

##### Consuming foods rich in iron

A negative relationship between IPV and consuming Fe-rich foods (plant and animal sources) was consistent in five of the eight countries analysed (except for Cambodia, Kenya and the Philippines, where associations did not reach the significance threshold, but were in the same direction). The pooled OR across the eight countries were also significant, indicating that IPV experience was associated with reduced consumption of Fe-rich foods. Past year sexual violence had the most extreme pooled association with dietary consumption of Fe: women exposed to sexual IPV had a 28 % decrease in the odds of consuming Fe-rich foods (OR: 0·72, 95 % CI: 0·53, 0·98). This equates to a 1·39 (95 % CI: 1·02, 1·89) factor increase in the odds of not consuming dietary Fe sources when women are exposed to sexual IPV.

In Cote D’Ivoire, Sierra Leone and Tajikistan, sexual IPV experience reduced the odds of consuming Fe-rich foods. In Nepal and Nigeria, physical and emotional IPV experience reduced the odds of consuming Fe-rich foods. The most extreme associations were noted in Cote D’Ivoire, Tajikistan and Sierra Leone. For example, Sierra Leonean women who had experienced sexual IPV were two times more likely to not consume Fe-rich foods (adjusted OR (aOR): 0·50, 95 % CI: 0·29, 0·88). A similar pattern was also noted among Tajik women (aOR: 0·51, 95 % CI: 0·27, 0·97). Overall, the pattern of findings for past-year IPV and dietary Fe consumption were robust in the sensitivity analysis, where only animal sources of Fe were considered (refer to online supplementary material, Supplemental Appendix F).

##### Consuming foods rich in vitamin A

Results concerning Vitamin A-rich foods were more varied, potentially reflecting local food environments and the availability of fruits and vegetables that are orange or yellow in colour. The pooled estimates were NS for all forms of IPV analysed. In Tajikistan, past year emotional IPV decreased the odds of consuming Vitamin A-rich foods (aOR: 0·70, 95 % CI: 0·52, 0·95). In Nigeria, past year emotional IPV reduced the odds of consuming Vitamin A-rich foods (aOR: 0·80, 95 % CI: 0·67, 0·94). In the Philippines, past year physical and sexual IPV were associated with reduced odds of consuming vitamin A-rich foods. Sexual IPV had the most extreme measure of association in the Philippines: the odds of consuming Vitamin A-rich foods were 48 % lower among women exposed to past year sexual IPV (aOR: 0·52, 95 % CI: 0·33, 0·83). This translates to a nearly two-fold increase in not consuming Vitamin A foods for Filipina women exposed to sexual IPV. Emotional IPV was marginally insignificant in the Philippines. Sierra Leone was the only country wherein results patterns ran contrary to our hypothesis: all forms of past year IPV increased the odds of consuming Vitamin A-rich foods.

### Discussion

We investigated the relationship between past-year IPV and women’s dietary intake of Fe and vitamin A-rich foods in eight LMIC. Cambodia was the only country where no forms of IPV were significantly associated with dietary Fe and vitamin A intake. In five countries (Cote D’Ivoire, Nepal, Nigeria, Sierra Leone and Tajikistan), at least one form of past year IPV was associated with reduced odds of consuming Fe-rich foods. The pooled estimates for emotional and sexual IPV and the consumption of Fe-rich foods were also significant (physical IPV was marginally insignificant). The strongest pooled association was between sexual violence exposure and dietary Fe intake. Results concerning the dietary consumption of Vitamin A were context-specific. While no pooled estimates of the Vitamin A outcome were significant, the most consistent and strong relationship between IPV and reduced dietary vitamin A consumption was noted in the Philippines. The impact of poor micronutrient intake from food sources is particularly important among women of reproductive age and pregnant/lactating women who may have poor use/adherence to supplements and face elevated IPV risks. In a context where health services are limited, food sources are the primary means of maintaining ideal Fe and Vitamin A levels.

Increasing the dietary and supplement intake of Vitamin A is relevant for countries with a high baseline level of night blindness among pregnant women^(10)^. In the second trimester and beyond (when nutritional demands are higher), night blindness signals moderate to severe Vitamin A deficiency that can affect fetal development^(10)^. In the Philippines, the most recent prevalence of night blindness among pregnant women is 8·6 %, which exceeds the threshold (> 5 %) of being a public health problem^(42)^. Regarding Fe-deficiency anaemia, women and girls living at higher altitudes may experience greater symptoms of Fe deficiency and dietary Fe needs. This is explained by the need to maintain higher haemoglobin levels to compensate for the lower oxygen tension. Additional challenges for maintaining haemoglobin exist in areas of endemic malaria. Thus, the consistent and robust findings regarding IPV and reduced dietary Fe intake are particularly relevant for contexts of elevated altitudes or endemic malaria.

Scholarship at the intersection of violence and nutrition illustrates potential mechanisms. One pathway is that IPV often occurs in contexts of high levels of controlling behaviours which act to impair women’s intake of nutrient-dense food sources, regardless of household food security^(43)^. Women experiencing IPV may thus be more likely to eat last and least compared to other household members. Gender inequitable beliefs and norms can lead to prioritising men’s intake of Fe-rich foods at the expense of women within a household^(43)^. A second pathway involves mental health. There are well-established links between IPV and mental health outcomes such as depression and post-traumatic stress disorder^(44)^, which can also result in poverty and food insecurity. Untreated depression can manifest in poor appetite and inadequate food intake, thereby contributing to poor intake of key nutrients such as Fe or vitamin A^(27)^. Survivors of sexual IPV also experience internalised and community stigma, which can limit access to more expensive food items such as animal protein sources. Additionally, physical IPV can result in traumatic injuries involving the brain, head and neck as well as direct oral and maxillofacial damage^(45)^. Such traumatic physical injuries can contribute to impaired micronutrient intake through poorer cognition, neurologic impairment, chronic jaw pain or diminished self-care practices^(46)^.

Future research directions include the longitudinal examination of nutrient intake impact following exposure to IPV both in the short term (months) and longer term (years). Such examination would also include biomarkers of nutrient intake and, for the Fe outcome, account for increased red blood cell turnover in settings where malarial exposure is also prevalent. Mixed methods studies can account for local variations in food access and diet practices. Future research may also consider the role of IPV frequency and severity to assess the potential for a dose–response relationship as well as exposure to overlapping forms of IPV on diet. Lastly, to assess whether women’s dietary intake is related to children’s dietary intake, mediation analyses can be conducted to develop a richer understanding of how IPV is related to intergenerational maternal–child health dipartites.

Importantly, this work provides further evidence for integrating IPV protections within health and nutrition services. Health and nutrition practitioners can refer to existing guidance on integrating strategies for preventing and responding to gender-based violence within their programmes^(1,47)^. For example, adding to ongoing nutrition assessments questions related to IPV risk, as relevant to nutrition-related programming, policies and communications^(1,47)^. These guidelines also recommend targeted screening for IPV based on known signs and symptoms such as mental and physical injuries^(1,47)^. Our results suggest additional considerations that can be added to current guidance. Routine blood testing for Fe levels and diagnosing pregnancy-related night blindness can be complemented by using the MDD-W tool to efficiently assess recent intake of foods rich in Fe and Vitamin A. Identification of women and girls who are not consuming foods rich in these nutrients can be a trigger for targeted screening for IPV.

The integration of IPV risk screening in nutrition and health services requires two important considerations: training needs and readiness for integration. To address training needs, UNICEF has developed a publicly available training course on mitigating gender-based violence risk for nutrition responders^(48)^. In addition, readiness for implementation requires examining whether risk assessments can be conducted confidentially and safely and whether specialised gender-based violence services are available. In contexts where violence screening is feasible, safe and confidential, the intake of Fe and vitamin A-rich foods, anaemia and night blindness can be used as additional indicators of possible exposure to IPV that may result in referral to gender-based violence services.

Differences between included countries, in terms of gender equality, food cultures and dietary diversity, may explain the different magnitude, significance and direction of results. For example, while all countries had existing legislation for domestic violence (criminal sanctions or protection orders), countries differed by year of enactment (the most recent being in 2022 in Cote D’Ivoire, 2016 in Kenya, 2013 in Tajikistan)^(49)^. This may reflect differing levels of recognising IPV as a human rights violation and reduced opportunities for survivors to seek aid and care. Generally, as GDP per capita increases so too does the consumption of Fe-rich food sources from animal protein^(50)^. The Philippines was the country with the highest GDP per capita^(50)^. This could explain the lack of relationship between IPV and dietary consumption of Fe sources.

One finding was contrary to our original hypothesis. In Sierra Leone, IPV was associated with a greater odds of consuming Vitamin A-rich fruits/vegetables. The 2019 Sierra Leonean DHS was conducted between May and August 2019, corresponding to the ‘lean season,’ wherein households are more prone to hunger due to increased food prices and limited food supply. The positive relationship between IPV and consumption of Vitamin A-rich foods could reflect the overconsumption of vitamin A-rich starchy staple vegetables (i.e. sweet potatoes, cassava) during the lean period. This pattern may be more pronounced among women exposed to violence. The sensitivity analysis also revealed that only the two lowest household wealth quintiles (poorest and poorer) were more likely to consume Vitamin A-rich vegetables (including sweet potato and cassava, etc.,). Sierra Leone was also unique from other countries given that IPV reporting overall was the highest and increased with each increasing household wealth quintile. These findings may suggest context-specific dynamics that are not shared in other countries: a context where IPV prevalence is generally high and remains high across wealth quintiles.

Regarding strengths, this work is the first to examine the population-level relationship between IPV and women’s dietary intake of key nutrients using nationally representative data in LMIC. We leveraged comparable indicators from multiple countries using appropriate population-based measurement indicators for IPV and dietary diversity. Our findings can enable researchers, practitioners and policymakers to draw both within and between country conclusions. Nonetheless, the present work has important limitations.

We are limited by the cross-sectional nature of the DHS and the indicators that were originally collected, thereby reducing our ability to examine causality. While the past year’s recall period of IPV was longer than the recall period of dietary intake (past 24 h), measurement limitations like recall error and telescoping bias (wherein recent events are recalled as happening in the more distant past and distant events are recalled as happening in more recent time) prevent the correct temporal positioning of the exposure and outcome variables. Thus, we cannot be sure that IPV preceded dietary intake patterns.

Diet was measured using the MDD-W tool, which captures food intake only from the prior day, providing population-level estimates of diet patterns, but not individual estimates of habitual diet, exact micro-nutrient intake, portion size or numbers of servings eaten. The MDD-W list-based recall method asks participants to recall consumption of common foods (in the past 24 h) from a pre-defined food list that is adapted to a specific country^(41)^. Thus, only a proxy marker for Fe nutritional status (i.e. consumption of foods containing Fe or vitamin A) could be constructed. These DHS surveys were also not implemented to permit capturing seasonal food variation.

Finally, several of our data sets included the first data collection completed after the pandemic: Cambodia, Ivory Coast, Kenya, Nepal, Philippines and since the war in Ukraine had begun: Cote D’Ivoire, Kenya, Nepal, Philippines). Both geo-political events severely affected food supplies in many LMIC countries. Further, the COVID-19 pandemic was linked to increased IPV perpetration^(51)^ and greater household food insecurity^(52)^. Thus, the pandemic may have led to worsened IPV and dietary intake of micronutrient-rich foods.

#### Conclusion

This work estimated the total relationship between IPV and intake of micronutrient-rich foods among women of reproductive age in eight LMIC. Results indicate robust and consistent relationships between IPV and reduced intake of dietary Fe sources. National variations were noted in the relationship between IPV and dietary Vitamin A consumption, with the Philippines having the most consistent association between IPV and reduced Vitamin A consumption. This research is an important stepping stone for understanding how IPV is associated with reduced health among women and extends the nutrition and gender-based violence nexus by focusing solely on women’s nutrition.

## Supporting information

Vahedi et al. supplementary material Vahedi et al. supplementary material

## Supplementary material

For supplementary material accompanying this paper visit https://doi.org/10.1017/S1368980025000539
