# Supplementary material for: Intimate partner violence and reduced dietary iron and vitamin A intake: a population analysis of nationally representative data from eight low- and middle-income countries
Source: Public Health Nutr. 2025 Apr 14;28(1):e87. doi: 10.1017/S1368980025000539 (PMC12100563; doi:10.1017/S1368980025000539)
Supplement: Vahedi et al. supplementary material [file S1368980025000539sup001.docx]

Supplementary Appendix

**Supplementary Appendix A: Sample DHS Questionnaires**

The Phase 8 model women’s questionnaire can be accessed here: <https://dhsprogram.com/pubs/pdf/DHSQ8/DHS8_Womans_QRE_EN_14Feb2023_DHSQ8.pdf>

The sample domestic violence module included in the DHS can be accessed here: <https://www.dhsprogram.com/pubs/pdf/DHSQMP/domestic_violence_module.pdf.pdf>

Sample Minimum Dietary Diversity for Women tool used in the 2022 Nepal DHS (page 680, Supplementary AppendixE): <https://dhsprogram.com/pubs/pdf/FR379/FR379.pdf>

**Supplementary Appendix B. Directed Acyclic Graph Guiding Covariate Selection**


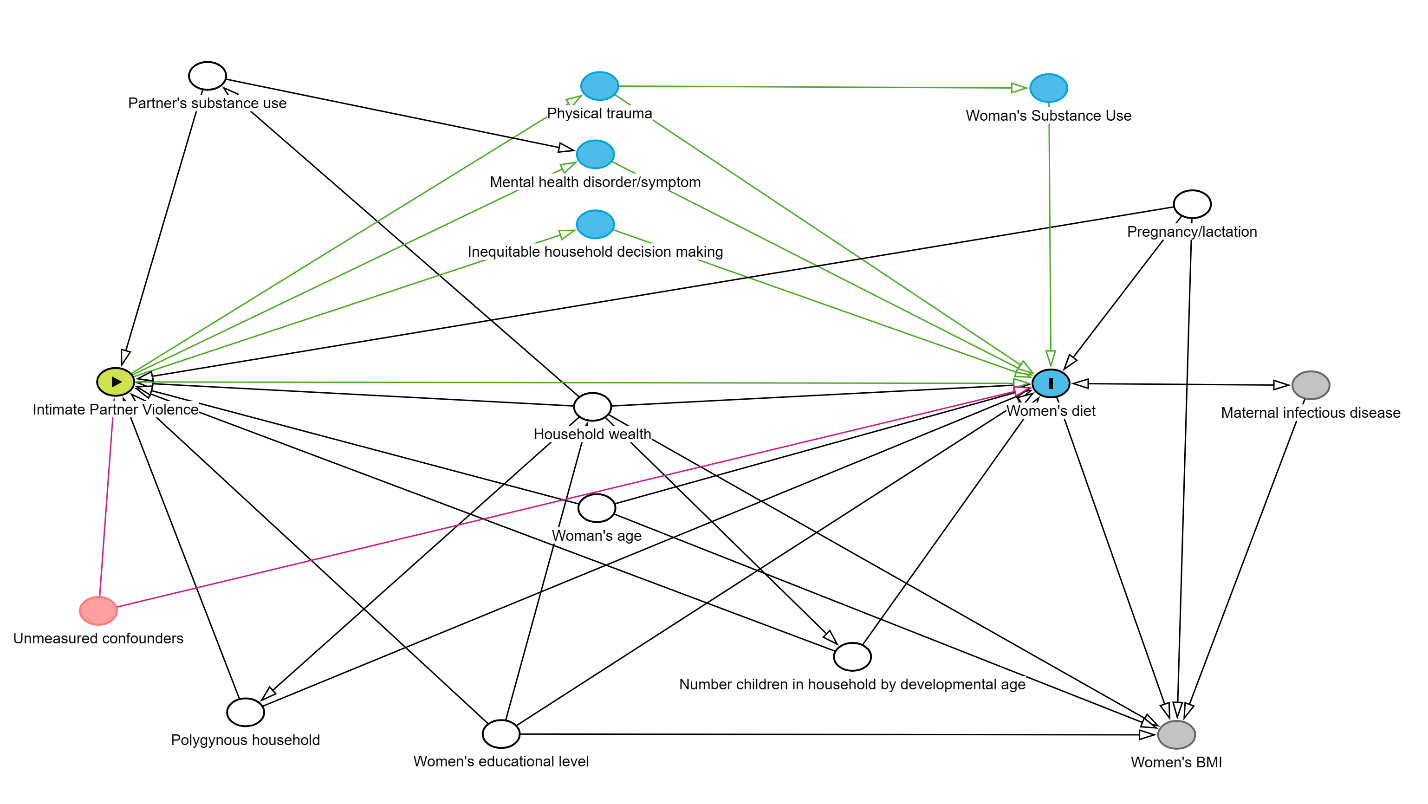


Infectious or chronic disease among women

Figure 1. Directed acyclic graph depicting the total effect of IPV and women’s diet, among women of reproductive age (15-49 years) currently at risk of experiencing IPV and living with abusers.

**Legend**

**Exposure**: Intimate partner violence (physical, emotional or sexual forms)

**Outcome:** Women’s diet (consumption of foods rich in iron and vitamin A)

**Mediators**:

- Physical traumas (broken bones, maxillofacial trauma, bruising, internal bleeding)
- Women’s substance use (alcohol, prescription opioids, or illicit drugs)
- Mental health disorders/symptoms (depression, anxiety, post-traumatic stress disorder, somatization)
- Inequitable household decision making (women’s reduced autonomy concerning resource use)

**Confounders**:

- Househol wealth (a measure of socio-economic status)
- Woman’s age (chronological age)
- Number of children in the household by developmental age (dependent children in the home during infancy, early, middle and late childhood and adolescent periods of development)
- Women’s education level
- Polygynous household (household practices polygyny, meaning there are multiple wives per husband)
- Partner’s substance use (alcohol, prescription opioids, or illicit drugs)
- Pregnancy/lactation (whether woman is currently pregnant or breastfeeding)

**Colliders:**

- Infectious or chronic disease among women (infectious diseases: malaria, HIV, respiratory infections, cholera; chronic diseases: type 2 diabetes, high blood pressure and/or cholesterol, cancer)
- Women’s BMI (body mass index)

The research team developed the directed acyclic graph (DAG), depicted in Figure 1, based on available literature. The DAG should be interpreted within the context of individual countries. The green paths depict the five causal pathways between IPV and women’s diet. The blue circles represent mediators between the IPV exposure and the women’s diet outcomes. The white circles depict confounding variables that we adjusted for in the statistical analyses. The pink circle represents unmeasured confounders that we could not adjust for analytically and the potentially biasing paths. The grey circles depict other variables, such as colliders, which should not be controlled for. All estimated parameters in the present research represent total adjusted effects between IPV and diet (representing all causal paths). To estimate the total estimated effect, we controlled for all confounding variables based on the above DAG: Household wealth, number children in household by age, partner's substance use, polygynous household, pregnancy/lactation, woman's age, women's educational level. In Cambodia and the Philippines, questions about polygyny were relevant and thus not surveyed. In the next sections we draw on the literature to justify the inclusion of the confounders, colliders, and mediators presented in Figure 2.

**Explanation of the mediators:** IPV can magnify physical trauma, mental health disorders/symptoms (depression, post-traumatic stress disorder, anxiety, somatization), substance use, and inequitable household decision making. Each consequence of IPV has implications for women’s diet and intra-household food allocation. For example, depression often manifests with a loss of appetite and symptoms of depression and anxiety can manifest in differing food choices.[1–4] Second, poor mental health may also contribute to women’s increased food insecurity by impacting her ability to carry out activities of daily living including obtaining and utilizing (preparing) food, as well as activities that may generate income. This can suppress the number and types of foods available for intake and women’s access to a diverse profile of food items.[5]

**Explanation of the confounders:** Several measured and unmeasured confounders affect the estimation of the relationship between women’s IPV and the dietary intake. We include household wealth and women’s education as constructs related to socio-economic status since lower socio-economic status increases risk of IPV [6] and can negatively impact household food affordability [7]. Also included is women’s age, as the risk for past year IPV is highest in early adulthood and adolescence [8]. Age can also affect food preferences and food allocation within a home [9]. Further, since polygynous families co-reside and may encounter competition for resources, we noted potential links with lower access to food and elevated IPV [10]. Caretaking children at distinct stages in development (infancy, early childhood, middle childhood, early adolescence) requires different feeding practices that vary by frequency and degree of care/attention [11]. As an example, breastfeeding and feeding/preparing food for children in early childhood could require more time and labour (resulting in women’s neglect of their own diet), compared to children at later stages of development. The risk and severity of IPV can also increase during and shortly after pregnancy [12]. The husband/partner’s substance use is a predictor of IPV and can further engender worsened mental health adversities for women (a hypothesized mediator) [13].

**Explanation of colliders:** Women’s BMI is positioned as a collider. Several confounders (i.e., household wealth, infectious disease, women’s education, and diet) can impact BMI. Similarly, we posited that maternal infectious and chronic disease can have a bi-directional relationship with diet: micronutrient deficiencies and diet quality overall can increase vulnerability and may worsen prognosis of infectious and chronic diseases [14] [15]. Infectious and chronic diseases can in turn affect diet by requiring dietary alterations, suppressing appetite, and via symptoms like nausea and diarrhea.

**Supplementary Appendix C: Covariate Operationalization for Confounding Variables**

Household wealth: The household wealth index is constructed by the DHS using principal component analysis. Households are placed on a standardized scale of relative wealth and divided into ordinal wealth quintiles: poorest (referent), poor, middle, less poor, least poor. Household survey questions concerning ownership of consumer products such as televisions or vehicles, residence characteristics such as the flooring material, source of drinking water, and water and sanitation facilities among others are used to compute the household wealth quintiles. Households are assigned a weighted score for each owned asset. A summed score is then computed, and the sample is divided into quintiles of household wealth.

Women’s educational level: We constructed an ordinal variable for women’s highest completed educational level at the point of interview: no education (referent), primary school, and secondary school or higher. To create this variable we used the survey question asking: “What is the highest level your attended: primary, secondary, or higher?”. If respondents previously reported never attending school, they were coded as having no formal education. The only deviation from this operationalization were for Tajikistan and the Philippines, where on average women’s educational attainment was higher. Highest education for Tajikistan and the Philippines was operationalized as no education or primary school (referent), secondary, and higher than secondary to maximize the model fit.

Women’s age: Women’s chronological age in years (numerical) was derived from the survey question asking: “How old were you at your last birthday” and verifies using a woman’s reported month and year of birth.

Husband’s degree of alcohol consumption: We constructed an ordinal variable to capture the degree to which a woman’s husband/partner consumes alcohol: does not consume alcohol (referent), consumes alcohol but is never drunk, consumes alcohol and is sometimes drunk, consumes alcohol and is often drunk. Respondents who indicated ‘no’ to the question “Does your husband/partner drink alcohol” were coded as ‘does not consume alcohol’. Respondents who indicated ‘yes’ were then coded according to their responses to the question: “How often does he get drunk: often, only sometimes, or never?”. The only deviation was in Tajikistan wherein only 14 respondents reported their partner was ‘never drunk’ and this category was collapsed with ‘does not consume alcohol’ to maximize the model fit.

Polygyny: To capture polygyny, wherein a male spouse has more than one wife, we created a binary variable: not polygynous (referent) and polygynous. Women who were not married and married but not living in a polygynous relationship were coded as ‘not polygynous’. Women who reported their husband having more than one wife (or cohabitating with other women as if married) were coded as ‘polygamous’. The polygyny variable was created using the question: “Does your (husband/partner) have other wives or does he live with other women as if married: Yes, No.”

Pregnancy and breastfeeding status: A binary variable capturing a woman’s current pregnancy and lactation status was created using the following questions: “Are you pregnant now: Yes, No, Unsure” and “Are you still breastfeeding your most recently birthed child: Yes, No”. Women who indicated ‘yes’ to either question were coded as ‘currently pregnant or breastfeeding’ and women who indicated ‘no’ and/or ‘unsure’ to both questions were coded as ‘not currently pregnant or breastfeeding’ (referent).

Total number of live births: The total number of children ever born alive to a woman (numerical variable) was constructed using the survey questions inquiring about whether or not a woman had ever given birth, the total number of biological sons and daughters living with the respondents, and the total number of biological sons and daughters who are not living with the respondent, and the number of children born alive but died later.

Children under 2 years of age in the household*:* A binary variable indicating whether children under 2 years of age are living in the household was constructed: no children under 2 years living in the household (referent) and children under 2 years are living in the household. This variable was created using the survey roster of children each woman had ever given birth to, whether the children were currently living in the household, and their chronological age at the point of interview. If a woman reported none of her children were under 2 years of age and living in the household or if a woman had never given birth to a child, responses were coded as ‘no children under 2 years living in the household’. If a woman reported having at least one child who was under 2 years of age and living in the household, responses were coded as ‘children under 2 years are living in the household’.

Children between 2 and 5 years of age in the household: A binary variable indicating whether children between 2 and 5 years of age are living in the household was constructed: no children between 2 and 5 years living in the household (referent) and children between 2 and 5 years are living in the household. This variable was created using the roster of children each woman had ever given birth to, whether the children were currently living in the household, and their chronological age at the point of interview. If a woman reported none of her children between 2 and 5 years of age and living in the household or if a woman had never given birth to a child, responses were coded as ‘no children between 2 and 5 years living in the household’. If a woman reported having at least one child living in the household who was between 2 and 5 years, responses were coded as ‘children under between 2 and 5 years are living in the household’.

Children between 5 and 9 years of age in the household: A binary variable indicating whether children between 5 and 9 years are living in the household was constructed: no children between 5 and 9 years living in the household (referent) and children between 5 and 9 years are living in the household. This variable was created using the roster of children each woman had ever given birth to, whether the children were currently living in the household, and their chronological age at the point of interview. If a woman reported none of her children between 5 and 9 years and living in the household or if a woman had never given birth to a child, responses were coded as ‘no children between 5 and 9 years living in the household’. If a woman reported having at least one child living in the household who was between 2 and 5 years, responses were coded as ‘children under between 5 and 9 years are living in the household’.

Children between 10 and 13 years in the household: A binary variable indicating whether children between 10 and 13 years are living in the household was constructed: no children between 10 and 13 years living in the household (referent) and children between 10 and 13 years are living in the household. This variable was created using the roster of children each woman had ever given birth to, whether the children were currently living in the household, and their chronological age at the point of interview. If a woman reported none of her children between 10 and 13 years and living in the household or if a woman had never given birth to a child, responses were coded as ‘no children between 10 and 13 years living in the household’. If a woman reported having at least one child living in the household who was between 10 and 13 years, responses were coded as ‘children under between 10 and 13 years are living in the household’.

**Supplementary Appendix D: Results Tables for the Generalized Linear Models**

| **Outcome: Consuming iron rich foods** | | | | **Outcome: Consuming Vitamin A rich foods** | | | |
| --- | --- | --- | --- | --- | --- | --- | --- |
| **Country** | **Emotional IPV** | **Physical IPV** | **Sexual IPV** | **Country** | **Emotional IPV** | **Physical IPV** | **Sexual IPV** |
| **Year** | **aOR** | **aOR** | **aOR** | **Year** | **aOR** | **aOR** | **aOR** |
| **N** | **p-value** | **p-value** | **p-value** | **N** | **p-value** | **p-value** | **p-value** |
|  | **95% CI** | **95% CI** | **95% CI** |  | **95% CI** | **95% CI** | **95% CI** |
| **Cambodia** | 0·969 | 0·704 | 1·055 | **Cambodia** | 0·976 | 0·96 | 1·212 |
| 2021 | 0·94 | 0·56 | 0·96 | 2021 | 0·84 | 0·84 | 0·51 |
| N=5,640 | [0·427, 2·199] | [0·214, 2·315] | [0·147, 7·601] | N=5,636 | [0·771, 1·235] | [0·649, 1·419] | [0·683, 2·151] |
| **Cote D’Ivoire** | 0·827 | 0·736 | 0·437 | **Cote D’Ivoire** | 0·98 | 1·148 | 1·421 |
| 2022 | 0·25 | 0·13 | 0 | 2022 | 0·91 | 0·48 | 0·21 |
| N=3,656 | [0·596, 1·147] | [0·495, 1·094] | [0·262, 0·730] | N=3,656 | [0·682, 1·409] | [0·780, 1·690] | [0·821, 2·459] |
| **Kenya** | 1·037 | 1·04 | 1·103 | **Kenya** | 1·127 | 0·947 | 1·306 |
| 2022 | 0·68 | 0·64 | 0·4 | 2022 | 0·14 | 0·55 | 0·06 |
| N=10,758 | [0·874, 1·231] | [0·882, 1·226] | [0·877, 1·387] | N=10,750 | [0·963, 1·318] | [0·792, 1·133] | [0·988, 1·727] |
| **Nepal** | 0·657 | 0·719 | 0·895 | **Nepal** | 0·856 | 1·065 | 0·904 |
| 2022 | 0 | 0·02 | 0·63 | 2022 | 0·44 | 0·74 | 0·69 |
| N=4,179 | [0·511, 0·844] | [0·549, 0·943] | [0·570, 1·406] | N=4,178 | [0·576, 1·270] | [0·737, 1·540] | [0·547, 1·493] |
| **Nigeria** | 0·718 | 0·702 | 1·011 | **Nigeria** | 0·796 | 0·914 | 1·163 |
| N=8,313 | 0·01 | 0·03 | 0·96 | N=8,313 | 0·01 | 0·48 | 0·32 |
| 2018 | [0·564, 0·913] | [0·514, 0·959] | [0·681, 1·501] | 2018 | [0·674, 0·940] | [0·713, 1·171] | [0·866, 1·561] |
| **Philippines** | 0·667 | 1·123 | 0·488 | **Philippines** | 0·806 | 0·659 | 0·525 |
| 2022 | 0·12 | 0·8 | 0·16 | 2022 | 0·07 | 0·02 | 0·01 |
| N=12,278 | [0·399, 1·116] | [0·463, 2·723] | [0·180, 1·321] | N=12,267 | [0·637, 1·021] | [0·460, 0·945] | [0·332, 0·830] |
| **Sierra Leone** | 0·786 | 1·277 | 0·5 | **Sierra Leone** | 1·317 | 1·386 | 1·649 |
| 2019 | 0·31 | 0·3 | 0·02 | 2019 | 0 | 0 | 0·03 |
| N=3, 812 | [0·492, 1·255] | [0·805, 2·027] | [0·285, 0·876] | N=3, 812 | [1·098, 1·580] | [1·127, 1·705] | [1·057, 2·572] |
| **Tajikistan** | 0·862 | 0·819 | 0·507 | **Tajikistan** | 0·701 | 0·862 | 1·552 |
| 2017 | 0·35 | 0·16 | 0·04 | 2017 | 0·02 | 0·23 | 0·21 |
| N=4,800 | [0·633, 1·175] | [0·621, 1·079] | [0·265, 0·969] | N=4,800 | [0·519, 0·946] | [0·675, 1·102] | [0·783, 3·077] |

IPV=Intimate Partner Violence, aOR=adjusted Odds Ration, CI=Confidence Interval

**Supplementary Appendix E: Structural Equations for the Generalized Linear Modelling**

The adjusted structural model for the logit models computed is given by Equation 1, where the natural logarithm of the odds for each binary dietary outcome (consumption of iron-rich foods and consumption of vitamin A foods) is given by 𝑙𝑛 (𝑝𝑖/1-𝑝 𝑖) where p_i_ is the probability of meeting the diet requirement. The natural logarithmic odds of the outcome is a linear function of all independent variables, showing the effect of each intimate partner violence independent variable (past year physical intimate partner violence, sexual intimate partner violence, and emotional intimate partner violence, represented by 𝛽 1 in separate models) on the binary diet variables, while holding the covariates (X) and the vector of covariate regression parameters (B), that were informed by the DAG, constant.

Equation 1. Structural model for the Logit Models

$$\ln\left( \frac{p_{i}}{1-p_{i}} \right)=\beta_{0}+\beta_{1}x_{i}+\beta X$$

**Supplementary Appendix F: Sensitivity Analyses**

**Part 1: Effect Modification**

For the sensitivity analysis in Table 1, we further investigated findings that appeared contrary to our a-priori hypotheses. Using the Sierra Leone dataset, we included only vitamin A-rich vegetables (i.e., starchy staples) and regressed this outcome on any IPV exposure. We display the results stratified by household wealth (as opposed to controlling for household wealth). This analysis enabled us to analyze whether the relationship between ‘any IPV in the past year’ and intake of Vitamin A vegetables differed by household wealth.

Table 1. Sensitivity Analyses for Sierra Leone: Adjusted Relationship between any Past Year IPV and Consuming Vitamin A rich Vegetables, Stratified by Household Wealth.

|  | **Vegetables Rich in Vitamin A (binary) N=3,812** | | | | | | | | | |
| --- | --- | --- | --- | --- | --- | --- | --- | --- | --- | --- |
|  | **Richest** | | **Rich** | | **Middle** | | **Poor** | | **Poorest** | |
| **Any IPV in the past year** | | | | | | | | | | |
| aOR | 1·056 | | 1·231 | | 1·400 | | 1·625 | | 1·446 | |
| p-value | 0·829 | | 0·293 | | 0·084 | | 0·005 | | 0·046 | |
| 95% CI | 0·643 | 1·734 | 0·835 | 1·815 | 0·956 | 2·051 | 1·156 | 2·284 | 1·006 | 2·078 |

Note: All generalized linear models were adjusted for variables deemed to be confounders: women’s education, women’s age, husband’s degree of alcohol consumption, presence of a polygynous household (except for Cambodia and Philippines), current pregnancy and/or breastfeeding status, number of live births, presence of children in the household by developmental age (under 2 years, between 2 and 4 years, presence of children between 5 and 9 years, and presence of children 10 to 13 years). The stratified results were computed by decomposing the linear estimated coefficient for intimate partner violence exposure by the level of household wealth from the joint effects model.

**Part 2: Reporting of IPV by household wealth quintile**

As household wealth increased, we expected that IPV would decrease. We examined the predicted marginal probability of IPV reporting by household wealth quintile (refer to Table 5). In general, the probability of women reporting IPV decreased as household wealth increased. In fact, in all countries, the predicted probability of IPV was statistically less among the richest households compared to the poorest households. However, the only exception to this was Sierra Leone, where reporting of IPV was the highest overall and increased with each wealth quintile. In Sierra Leone, women from the richest households were statistically more likely to report violence compared to women from the poorest households. This sensitivity analysis yields two conclusions relative to our findings: (1) women’s reports of IPV do not deviate from what is theoretically expected in most countries analyzed except for Sierra Leone and (2) Sierra Leonan women had the highest burden of violence that remained consistently high among the wealth quintiles, with women from the highest wealth quintile reporting the most violence. These findings suggest that IPV is highly prevalent overall and across wealth quintiles in Sierra Leone.

Table 2. Probability of women (15-49 years), who are currently in a relationship, reporting past year IPV by level of household wealth.

| **Country** | **Household wealth quintile** | **Probability of reporting IPV (past year)** | **95% CI** | |  |
| --- | --- | --- | --- | --- | --- |
| Sierra Leone | poorest | 0·46 | 0·42 | 0·51 | * |
|  | poorer | 0·52 | 0·47 | 0·57 |  |
|  | richer | 0·53 | 0·48 | 0·58 |  |
|  | richest | 0·56 | 0·49 | 0·63 | * |
| Philippines | poorest | 0.11 | 0.09 | 0.12 | * |
|  | poorer | 0.11 | 0.09 | 0.13 |  |
|  | middle | 0.10 | 0.08 | 0.12 |  |
|  | rich | 0.06 | 0.05 | 0.08 |  |
|  | richest | 0.04 | 0.03 | 0.05 | * |
| Tajikistan | poorest | 0·31 | 0·26 | 0·36 | * |
|  | poorer | 0·30 | 0·25 | 0·36 |  |
|  | middle | 0·21 | 0·17 | 0·25 |  |
|  | richer | 0·22 | 0·19 | 0·25 |  |
|  | richest | 0·16 | 0·13 | 0·18 | * |
| Nigeria | poorest | 0·32 | 0·28 | 0·35 | * |
|  | poorer | 0·34 | 0·30 | 0·37 |  |
|  | middle | 0·33 | 0·30 | 0·35 |  |
|  | richer | 0·30 | 0·26 | 0·33 |  |
|  | richest | 0·21 | 0·18 | 0·24 | * |
| Nepal | poorest | 0·18 | 0·15 | 0·21 | * |
|  | poorer | 0·24 | 0·20 | 0·29 |  |
|  | middle | 0·20 | 0·16 | 0·24 |  |
|  | richer | 0·17 | 0·14 | 0·21 |  |
|  | richest | 0·09 | 0·07 | 0·12 | * |
| Kenya | poorest | 0·37 | 0·34 | 0·40 | * |
|  | poorer | 0·38 | 0·35 | 0·41 |  |
|  | middle | 0·36 | 0·33 | 0·39 |  |
|  | richer | 0·31 | 0·28 | 0·34 |  |
|  | richest | 0·22 | 0·19 | 0·25 | * |
| Cote D'Ivoire | poorest | 0·27 | 0·24 | 0·31 | * |
|  | poorer | 0·25 | 0·21 | 0·29 |  |
|  | middle | 0·27 | 0·22 | 0·32 |  |
|  | richer | 0·22 | 0·18 | 0·27 |  |
|  | richest | 0·16 | 0·11 | 0·22 | * |
| Cambodia | poorest | 0·21 | 0·18 | 0·24 | * |
|  | poorer | 0·16 | 0·13 | 0·19 |  |
|  | middle | 0·13 | 0·11 | 0·16 |  |
|  | richer | 0·12 | 0·10 | 0·15 |  |
|  | richest | 0·07 | 0·04 | 0·09 | * |
| Note: All probabilities are predicted marginal probabilities computed from a logistic regression with any past year IPV as the outcome predicted by ordinal household wealth. '*' denotes a statistically significant difference between predicted marginal probabilities comparing the poorest and richest wealth quintiles using the Wald Test. | | | | | |

**Part 3: Animal Sources of Iron**

We opted for a different operationalization for the iron-rich foods outcomes wherein only animal sources were considered (rather than animal and plant sources). Refer to Figure 1 to review the results. Our original findings concerning the pooled results are robust with the only exception being Nepal, wherein associations became insignificant with the use of the animal sources version of the iron-rich foods. However, Nepal does have a sizable proportion of vegetarians, so this result was anticipated.


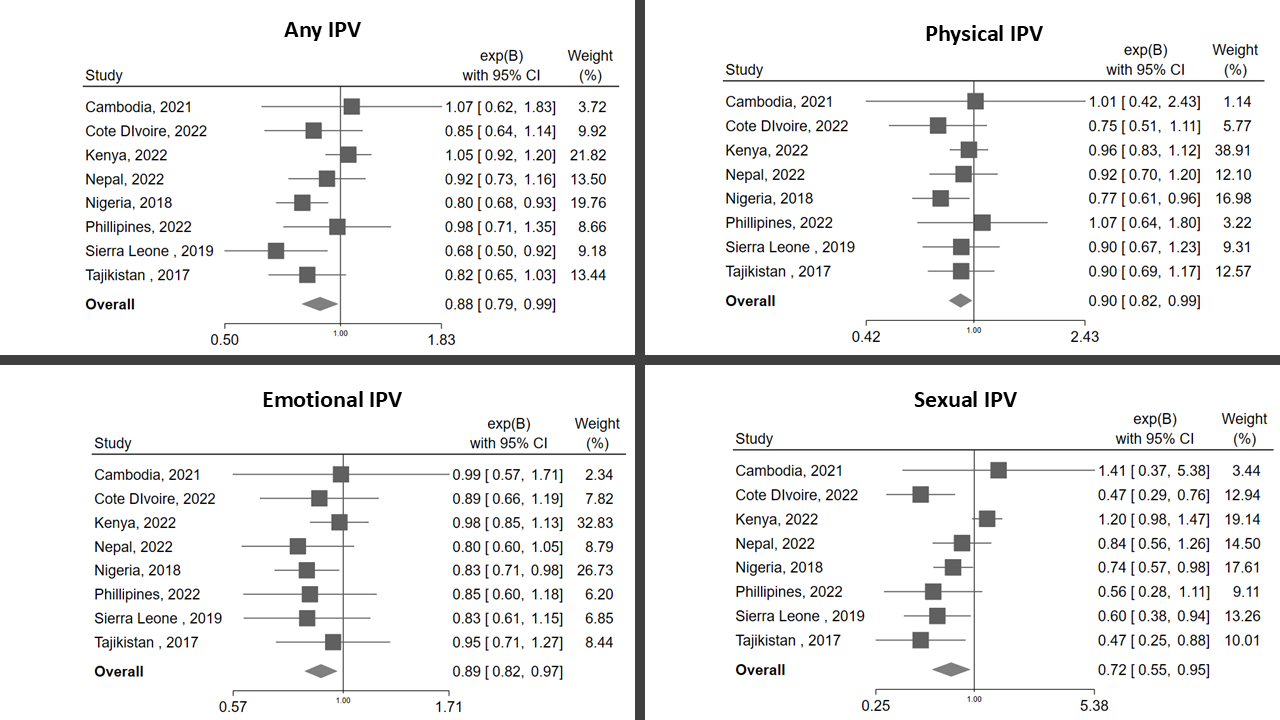


Figure 1. Sensitivity analysis for animal sources of dietary iron

**References**

1. Lang UE, Beglinger C, Schweinfurth N, Walter M, Borgwardt S. Nutritional Aspects of Depression. Cell Physiol Biochem. 2015;37(3):1029–43.

2. Marx W, Lane M, Hockey M, Aslam H, Berk M, Walder K, et al. Diet and depression: exploring the biological mechanisms of action. Mol Psychiatry. 2021;26(1):134–50.

3. Paans NPG, Gibson-Smith D, Bot M, Strien T van, Brouwer IA, Visser M, et al. Depression and eating styles are independently associated with dietary intake. Appetite. 2019;134:103–10.

4. Khan R, Waqas A, Bilal A, Mustehsan ZH, Omar J, Rahman A. Association of Maternal depression with diet: A systematic review. Asian J Psychiatry. 2020;52:102098.

5. McMichael AJ, McGuinness B, Lee J, Minh HV, Woodside JV, McEvoy CT. Food insecurity and brain health in adults: A systematic review. Crit Rev Food Sci Nutr. 2022;62(31):8728–43.

6. Ghoshal R, Douard AC, Sikder S, Roy N, Saulnier D. Risk and Protective Factors for IPV in Low- and Middle-Income Countries: A Systematic Review. J Aggress, Maltreatment Trauma. 2023;32(4):505–22.

7. Candler T, Costa S, Heys M, Costello A, Viner RM. Prevalence of Thinness in Adolescent Girls in Low- and Middle-Income Countries and Associations With Wealth, Food Security, and Inequality. J Adolesc Heal. 2017;60(4):447-454.e1.

8. Sardinha L, Maheu-Giroux M, Stöckl H, Meyer SR, García-Moreno C. Global, regional, and national prevalence estimates of physical or sexual, or both, intimate partner violence against women in 2018. Lancet [Internet]. 2022;399(10327):803–13. Available from: <https://doi.org/10.1016/S0140-6736(21)02664-7>

9. Verstraeten R, Leroy JL, Pieniak Z, Ochoa-Avilès A, Holdsworth M, Verbeke W, et al. Individual and Environmental Factors Influencing Adolescents’ Dietary Behavior in Low- and Middle-Income Settings. PLoS ONE. 2016;11(7):e0157744.

10. Ahinkorah BO. Polygyny and intimate partner violence in sub-Saharan Africa: Evidence from 16 cross-sectional demographic and health surveys. SSM - Popul Heal. 2021;13:100729.

11. Olson CM. Food Insecurity in Women: A Recipe for Unhealthy Trade-offs. Top Clin Nutr. 2005;20(4):321–8.

12. Brownridge DA, Taillieu TL, Tyler KA, Tiwari A, Chan KL, Santos SC. Pregnancy and Intimate Partner Violence: Risk Factors, Severity, and Health Effects. Violence Against Women. 2011;17(7):858–81.

13. Foran HM, O’Leary KD. Alcohol and intimate partner violence: A meta-analytic review. Clin Psychol Rev. 2008;28(7):1222–34.

14. Grant WB. Diet, Inflammation, and Infectious Diseases. Nutrients. 2023;15(13):2891.

15. Huttunen R, Syrjänen J. Obesity and the risk and outcome of infection. Int J Obes. 2013;37(3):333–40.
